# Deceased donor uterus transplantation: religious perceptions

**DOI:** 10.3389/frtra.2025.1536754

**Published:** 2025-02-28

**Authors:** Jana Pittman, Brigitte Gerstl, Elena Cavazzoni, Natasha Mireille Rogers, Mianna Lotz, Rebecca Deans

**Affiliations:** ^1^ New South Wales Department of Health, South Eastern Sydney Local Health District, Sydney, NSW, Australia; ^2^ Faculty of Medicine, School of Clinical Medicine, University of New South Wales, Sydney, NSW, Australia; ^3^ NSW Organ Tissue and Donation Service, Kogarah, NSW, Australia; ^4^ Discipline of Child and Adolescent Health, Faculty of Medicine and Health, School of Medicine, The University of Sydney, Sydney, NSW, Australia; ^5^ Department of Philosophy, Children’s Hospital at Westmead, Sydney, NSW, Australia; ^6^ Philosophy, Macquarie University, Sydney, NSW, Australia; ^7^ Faculty of Medicine, University of New South Wales, Sydney, NSW, Australia

**Keywords:** transplantation, infertility, uterus transplant, deceased donor, vascular composite allograft, uterus, public perception

## Abstract

**Background:**

Uterus transplant now offers an alternative deceased donation treatment option for women with uterine infertility. Limited research exists on religious opinions that may impact the addition of the uterus to current multi-organ deceased donor programs

**Objective:**

To explore the acceptability of uterus transplantation and deceased uterus donation across different religious groups.

**Design:**

A cross-sectional survey of 2,497 participants was conducted between October 2022 and January 2023 in NSW Australia. Australia is a culturally and religiously diverse nation with over 60% of people identifying with a religion, including Christianity (43%), Islam (3.2%), Buddhism (2.7%), Hinduism (2.4%). This survey captured awareness and attitudes towards deceased uterus donation. Descriptive statistics and regression analyses were used to explore factors influencing organ donation and next-of-kin perceptions.

**Results:**

A total of 2,497 respondents completed the survey. Christians had greater awareness of organ donation but were less likely to be registered donors, or consent to uterus donation. Those of Hindu faith were less likely to be registered organ donors. Next-of-kin from the Islamic faith were reluctant to consent to organ donation if the donor's pre-death wishes were unknown, and less likely to consent to uterus donation. Participants identifying as Buddhist had a higher awareness of uterus transplantation.

**Conclusion:**

Organ donor awareness and consent rates varied across religious groups, including for uterus donation. Differences may stem from varying beliefs about bodily integrity, and reproductive rights, which may influence attitudes toward uterus donation. Tailored culturally and linguistically sensitive educational campaigns should address the unique aspects of uterus donation.

## Introduction

Uterus transplantation (UTx) represents a significant advancement in reproductive medicine for women diagnosed with uterine infertility (UFI). This innovative procedure provides women with either an absent or dysfunctional uterus the chance to experience gestational motherhood. To date, over 80 uterus transplants have been carried out globally, resulting in over 40 successful live births (1, 2). Most UTx procedures have utilized living donors (elective donor hysterectomy), facilitating optimal surgical planning and greater graft quality (1, 3, 4). However, the success of the living donor approach is tempered by a 17% rate of major postoperative complications associated with living donors, as assessed by the Clavien–Dindo system (2, 5). Complications in living donors predominantly affect the urinary system, with issues such as hydronephrosis, utero-vaginal fistulas, and altered voiding sensation (2). To date there have been no fatalities reported in living donor UTx operations, however as with any major abdominal surgical procedure, this risk exists. The documented mortality rates for living kidney donation are 0.03% and 0.2% for liver donation (6, 7). A radical hysterectomy carries a mortality rate of 0.01%–0.03% (8). Vesicovaginal and ureterovaginal fistulas following radical hysterectomy occur in 0.9%–2% (9). A deceased donor program mitigates this morbidity and mortality risk for living donors. This research team has ethical approval to conduct living and deceased donor UTx clinical trials in Australia (2019/ETH13038, ACTRN12622000917730), however, the uterus has not yet been included in the Australian organ donor program.

Integrating uterus donation into any current multi-organ donor framework presents complex ethical, religious and cultural challenges. The uterus is a non-life-saving, and temporary transplant, limited to reduce exposure to immunosuppression (hysterectomy completed after 5–7 years, or once the recipient deems her family complete) (2). There is also the added complexity of the uterus being a reproductive organ, unlike the kidney or liver. Currently, there is a scarcity of publications that specifically examine how UTx aligns with the doctrinal views of individual faiths, or studies around the cross-cultural public acceptability of UTx (10). Varying religious beliefs may influence the acceptability of uterus donation and the consent process. Broadly, the Buddhist faith does not place explicit doctrinal emphasis on fertility, with its teachings often prioritizing the alleviation of suffering, which may make UTx a favourable option. Conversely, Orthodox Jewish perspectives place significant emphasis on the sanctity of reproductive organs. Catholic officials may consider the procedure morally problematic due to its reliance on assisted reproduction technology, which is viewed as separating procreation from the “conjugal act”, however, studies show that people of broader Christian faith have some of the highest rates of organ donation (11). Faith or cultural beliefs may influence the acceptability of uterus donation and the consent process. Understanding public perspectives on the acceptability of uterus donation is paramount prior to considering including the uterus in any current multi-organ donation program.

Australia is a multicultural society, with nearly half the population born outside of Australia, or having a parent born in another country (12). Additionally, Australia is religiously diverse, with over 60% of the population identifying with a religion, including but not limited to Christianity (43%), Islam (3.2%), Buddhism (2.7%), Hinduism (2.4%) (12). This diversity positions this study uniquely to explore how faith influences attitudes toward the inclusion of the uterus in existing multi-organ donation programs worldwide. Insights gained from this research may provide valuable guidance for developing educational strategies that respect diverse beliefs while promoting awareness of the potential benefits of UTx. Acknowledging and addressing the religious dimensions surrounding organ donation, including the uterus, is essential for ensuring a safe and culturally sensitive UTx program.

## Materials and methods

This study was approved by the University of New South Wales Human Research Ethics Committee (HC220047) and reviewed by the New South Wales, Organ and Tissue Donation Service (OTDS). A cross-sectional survey was administered to a diverse sample of 2,497 participants from varying religious and cultural backgrounds between October 2022 and January 2023. Participants were asked about their awareness of organ donation, their willingness to donate, and their views on UTx specifically. Participants were Australian residents or citizens, and aged 18 years and over. Recruitment was via a Qualtrics database using a single advertisement on Instagram. After providing informed consent, participants anonymously completed a 37-item survey via a secure, internet-based platform.

Demographic data included age, gender, religion, relationship status, ethnicity, highest level of education, employment status and household income. Participants were asked general questions regarding organ donation such as “*are you a registered organ donor?”,* and if not *“Would you consider donating your organs’.* Responses were categorized into three groups: “yes”, “unsure”, and “definitely no” to organ donation. These groups were then compared on their religious affiliation in response to specific questions relating to uterus donation (Supplementary Table S1).

Descriptive statistics were utilized, with frequencies and proportions calculated for categorical variables, and medians reported for continuous variables. Group comparisons were conducted using Chi-squared (*x*^2^) test or Fisher's exact test for categorical variables. Odds ratios were derived from regression analyses to explore associations between demographic factors and organ donation preference. Variables used in the model included religion (Islam, Buddhism, Hinduism, Judaism, no religion and not specified); gender (male vs. female); relationship status at the time of completing the questionnaire (single vs. relationship); and education (high school/trade vs. university degree). Statistical significance was set at *p* < 0.05 for all analyses. The data were analysed using STATA 18.0 (StataCorp, College Station, TX, USA) (13).

## Results

A total of 2,497 respondents completed the survey, with a mean age of 46yrs (range 18–84 years). See Table 1 for ethnic and gender demographic breakdown.

**Table 1:** Characteristics of the study population.

Demographics	Total *n (%)*
Gender	
Female	1,446 (57.9)
Male	1,030 (41.3)
Decline to answer/other	21 (0.8)
Religion	
No religion	1,277 (51.1)
Christian denomination	964 (38.6)
Hinduism	46 (1.8)
Islam	55 (2.2)
Buddhism	42 (1.7)
Judaism	15 (0.6)
Other	98 (3.9)
Ethnicity	
White	1,994 (78.9)
Asian	194 (7.8)
Indigenous Australian or Torres Strait Islander	121 (4.9)
Middle Eastern	30 (1.2)
Other	158 (6.3)

Overall, 1,614/2,497 (64.6%) respondents were aware that deceased organ donation was an option after death. Christian people were significantly more likely to report knowledge of organ donation (OR: 1.28, 95%CI: 1.02–1.61, *p* = 0.03). Pre-death “donor registration”, was significantly less likely in participants identifying as Christian (OR: 0.79, 95% CI: 0.67–0.93, *p* = 0.004), Hindu (OR: 0.51, 95% CI: 0.28–0.95, *p* = 0.03), or Islamic (OR: 0.51, 95% CI: 0.29–0.90, *p* = 0.02), compared to those who reported having no religion. Overall, 1,632/2,497 (65.3%) indicated they would consent to organ donation, even if unregistered. Of those not consenting, no significant differences were observed between specific religious beliefs and the likelihood of consenting to be an organ donor.

Participants were asked about “acting as next of kin”. 1,531/2,497 (61.3%) stated they would agree to donation regardless of whether they knew the donor's pre-death wishes. Participants reluctant to provide consent for next of kin donation were significantly more likely to be of Islamic faith, compared to those with other, or no religious affiliations (OR: 5.12, 95% CI:2.58–10.15, *p* < 0.001).

When those participants who were aware of overall organ transplant were asked whether they knew of *“uterus transplant”*, 533/1,614 (33.0%) answered “yes”. Participants identifying as Buddhist (OR: 3.48, 95%CI: 1.88–6.47, *p* = 0.004) or Islamic (OR: 2.64, 95%CI: 1.51–4.61, *p* < 0.001) exhibited an increased awareness of UTx. When participants were asked if they would “consent” to uterus donation as the designated next-of-kin, 2,304 participants answered with 1,032/2,304 (44.8%) indicating they would consent, and 355/2,304 (15.4%) would only consent if they were aware of the donors *“pre-death wishes.”* A further 749/2,304 (32.5%) remained unsure. Individuals identifying as Christian (OR: 0.64, 95% CI: 0.45–0.89, *p* = 0.01) or Islamic (OR: 0.44, 95% CI: 0.20–0.98, *p* = 0.04) were less likely to consent to uterus donation.

## Discussion

Deceased organ donor programs worldwide operate on varying frameworks that influence the allocation and acceptance of organ donations. This study revealed several insights into the awareness and acceptance of organ donation, including UTx, across different religious groups in Australia.

In many countries, individuals can register their wish to become an organ donor, indicating their willingness to donate their organs posthumously (14, 15). However, in the majority of cases, the final decision remains with the senior available next-of-kin (SaNOK), whose perspective on organ donation may be influenced by cultural, religious, or emotional factors (15). Language-specific cross-cultural resources exist to educate religious and linguistically diverse people on organ and tissue donation (16). This study was conducted in Australia, where 35% of the population are registered donors, and 55% of families consent to proceed with organ donation when approached in critical care settings (17) Wide variation exists within ethnically diverse groups, with 69% of Australian-born residents consenting, 73% among Southern and Central Asia (India), but only 27% among Southern and Eastern Europe (Greek), 25% among Southeast Asian (Filipino) and 12% among North-East Asian (Chinese) (18). Furthermore, registration and family discussion have a direct impact on donation consent, where consent decreases to 40% when families are unaware of the deceased's wishes regarding organ donation (17, 18).

In this study, participants identifying as Islamic and Christian reported greater awareness of organ donation but no significant increase in levels of agreeability towards becoming a donor or pre-death donor registration, compared with other religions and those with no religious affiliation. This is in keeping with Ralph et al. (19) who explored attitudes and beliefs around deceased organ and tissue donation in an Arabic-speaking Australian cohort, where currently donor rates are only 0.07%, their participants reported “concern and skepticism” towards the donation process. In 1995, the Muslim Law (Sharia) Council UK issued a fatwa declaring that organ donation is permissible (11, 18, 20, 21). This was re-affirmed in 2019, when Mufti Muhammad Zubair Butt acknowledged this permissibility but reported a significant difference in his view, arguing that organs should only be taken after cardiac death, in contrast with the initial ruling allowing removal after brainstem death (18, 20, 21). Conversely, previous international research suggests higher rates of pre-death donor registration and donor consent in countries where Christianity is the predominant religion (i.e., United States and European Union) (11, 22), indicating that some Christian communities may have reservations. Notably, most Christian denominations have publicly endorsed organ donation as an act of 'selflessness', and previous Catholic Popes have publicly stated they carried donor cards (11).

Amongst this study cohort, those of Hindu faith were less likely to be registered donors, contrasting with “DonateLife” (Australia) (2016) report that people from India had high (73%) next-of-kin consent rates. Hindu beliefs are shaped by the concepts of Dharma (duty), Karma (actions), and Atman (soul), and international studies report positive views on organ donation as an ethical duty, aligning with the principle of saving lives (22).

Concerning UTx, there remains limited published data examining how UTx aligns with the doctrinal views of individual faiths (10). In 2024, De Graca et al. (10) explored current religious conversations in reproductive medicine, including positions on surrogacy, adoption and, if known, UTx. They reported that most religious groups do not currently have official positions on UTx and that acceptability varied widely within groups, likely reflecting the fact that different faiths hold varying views on the sanctity of the body and the act of organ donation (10).

Although most participants in our study did not specify a religious affiliation, we found a significant association did exist between religious affiliation and awareness of UTx. Participants identifying as Buddhist (*p* = 0.004) and Islamic (*p* < 0.001) were significantly more likely to be aware of UTx compared to those with other or no religious affiliations. This heightened awareness may stem from the unique cultural and religious contexts that place a high value on fertility, family continuity, and reproductive health, particularly in the Islamic faith. It is important to note that while Islamic law generally prohibits surrogacy due to concerns about lineage and inheritance, UTx may be viewed differently within Islamic jurisprudence (23). In many Islamic communities, the desire for biological children is often tied to religious and familial expectations (10, 24, 25). While Buddhism does not place explicit doctrinal emphasis on fertility, its teachings often prioritize the alleviation of suffering, which could make UTx a favorable option in cases of infertility. Orthodox Jewish perspectives, however, place significant emphasis on the sanctity of reproductive organs, raising concerns over the halachic acceptability of such donations, particularly regarding physical contact with reproductive organs post-transplant. Furthermore, some Catholic viewpoints consider the procedure morally problematic due to its reliance on assisted reproduction technology, which is viewed as separating procreation from the “conjugal act” (26). These religious nuances could complicate the feasibility of uterus donation, particularly in obtaining timely consent from families.

This research reports religious differences in perspectives for uterus donation. It highlights the importance of ensuring families would have the choice to exclude the uterus from the donation process if UTx was translated into clinical care. It highlights the importance of offering families the choice to exclude the uterus from the donation process. In Australia, organ donation follows a selective approach, where families may consent to certain organs (e.g., liver or kidneys) but decline others (e.g., heart). While UTx is still in the clinical research phase, if the uterus were to be included in any multi-organ donor programs, decisions regarding its inclusion or exclusion would be made on a case-by-case basis, considering the deceased's wishes, family preferences, and medical suitability (27). Transparency throughout the consent process is vital to preserving the integrity of informed consent and upholding ethical standards in organ donation practices (28).

To enhance public education and awareness initiatives, future research should focus on developing targeted outreach programs that address the specific cultural and religious beliefs surrounding organ donation. This could involve conducting qualitative studies to better understand the unique barriers and facilitators to organ donation within different cultural contexts. Additionally, longitudinal studies are needed to assess the effectiveness of these educational interventions in improving organ donation rates among various religious and cultural groups. A key strategy for outreach could involve collaborating with religious leaders and community organisations to create culturally sensitive educational campaigns. These campaigns should aim to address the specific concerns and misconceptions of each group, using clear and accessible language. Dissemination of this information through community centres, places of worship, and social media platforms would maximise their reach and ensure they resonate with diverse populations. Lastly, we suggest that future research explore the potential of social media and digital platforms as tools for disseminating accurate, culturally sensitive information about organ donation and uterus donation. Xiao-Ya et al. (29)indicated that social media content can significantly influence public attitudes, suggesting that campaigns tailored to specific communities could play a key role in enhancing acceptance and awareness of organ donation overall. By incorporating these strategies, future efforts can better address the diverse needs of religious and cultural groups, improving the overall success of organ donation programs, including UTx.

Overall differences seen in our cohort, compared with other studies, highlight the potentially nuanced differences in donor registration and attitudes toward organ donation between countries, influenced by local cultural and social contexts, even within religiously similar groups. This highlights the need for ongoing research and community consultation in countries considering adoption of deceased donor UTx.

## Conclusion

Organ donor awareness and consent rates varied across religious groups, particularly for uterus donation. This difference may stem from varying beliefs about bodily integrity, and reproductive rights, which may influence attitudes toward uterus donation. Tailored culturally and linguistically sensitive educational campaigns should be created to address the unique aspects of uterus donation.

## Data Availability

The raw data supporting the conclusions of this article will be made available by the authors, without undue reservation.
