# Supplementary material for: Deceased donor uterus transplantation: religious perceptions
Source: Front Transplant. 2025 Feb 28;4:1536754. doi: 10.3389/frtra.2025.1536754 (PMC11906711; doi:10.3389/frtra.2025.1536754)
Supplement: Supplementary file 1 [file Table1.pdf]

Supplementary Table 1

Questionnaire: Deceased Donor Uterus Transplantation: Religious Perceptions

Question number	Deceased Donor Uterus Transplantation: Religious Perceptions- Questionnaire*
Q1	What is your current age?<br>a) 18-30 years<br>b) 31-45 years<br>c) 46-60 years<br>d) 61 years and over
Q2	How do you describe your gender?<br>a) Man/male<br>b) Woman/female<br>c) Non-binary<br>d) I use a different term [TEXT box]<br>e) Prefer not to answer
Q3	What is the highest level of education you completed?<br>a) High school<br>b) Diploma or trade certificate<br>c) Bachelor's degree<br>d) Postgraduate degree<br>e) Masters<br>f) Doctorate
Q4	Are you currently employed?<br>a) Working (paid or voluntary)<br>b) Not formally working (Looking after family member/care role)<br>c) Not working (looking for work)<br>d) Not working (unable)<br>e) Not working (retired<br>f) Prefer not to answer<br>g) Other (please specify) TEXT ANSWER
Q9	Please specify your ethnicity:<br>a) Caucasian<br>b) Indigenous Australian or Torres Strait Islander<br>c) Asian<br>d) Middle Eastern<br>e) Mixed race<br>f) Other (please specify)<br>g) I prefer not to say
Q11	Do you follow any religious practices?<br>a) Christian denomination<br>b) Islam<br>c) Buddhism<br>d) Hinduism<br>e) Judaism<br>f) No religion<br>g) Other (please specify) TEXT BOX
Q12	Relationship status<br>a) Single

	b) Defacto<br>c) Married<br>d) Divorced or separated<br>e) Widowed<br>f) I prefer not to say
Q14	Are you aware of how organ donation occurs in Australia (Donate Life)?<br>a) Yes<br>b) No<br>c) Unsure
Q17	Are you a registered organ donor?<br>a) Yes<br>b) No
Q18	Would you consent to be an organ donor?<br>a) Yes<br>b) Definitely not<br>c) Maybe/Unsure
Q 23	Would you consent to donation for your parent, spouse, sibling, or friend if you were the senior available next of kin (the person making decisions about donation)?<br>a) Yes, regardless even I did not know their wishes as donation helps save lives<br>b) Yes, but only if I knew their wishes (i.e. on the donor registry or have discussed with them)<br>c) No, even if they were on the donor registry, I do not believe in organ donation
Q 24	Are you aware of uterus transplant?<br>a) Yes<br>b) No
Q 27	In the event of a parent, spouse, or sibling's death, where you are the senior next of kin (the person making decisions about donation), would you allow the uterus to be donated?<br>a) Yes, but only if all the other organs are being donated too, not alone<br>b) Yes, even if the other organs could not be donated<br>c) Unsure<br>d) No

**This study formed part of an extensive questionnaire on organ transplantation. Only relevant questions to this paper 'Deceased Donor Uterus Transplantation: Religious Perception' have been included.*
